# Holographic Grating Enhancement Induced by a Dual-Photo-Initiator System in PMMA Substrate Polymers

**DOI:** 10.3390/polym16010126

**Published:** 2023-12-30

**Authors:** Peiyao Wang, Xiudong Sun, Peng Liu

**Affiliations:** 1 School of Physics and Electronic Engineering, Sichuan Normal University, Chengdu 610101, China; prairiee@foxmail.com; 2 Institute of Modern Optics, School of Physics, Key Laboratory of Micro-Nano Optoelectronic Information System, Ministry of Industry and Information Technology, Key Laboratory of Micro-Optics and Photonic Technology of Heilongjiang Province, Harbin Institute of Technology, Harbin 150001, China; xdsun@hit.edu.cn; 3 Collaborative Innovation Center of Extreme Optics, Shanxi University, Taiyuan 030006, China

**Keywords:** dual-photo-initiator system, dark diffusion, photopolymerization, diffraction efficiency, nonlocal polymerization-driven diffusion

## Abstract

Polymer systems induced by the reaction between monomers and photo-initiators play a crucial role in the formation of volume-phase gratings. In this paper, we fabricated a dual-photo-initiator photopolymer by doping EY (Eosin Yellow) molecules into a TI (Titanocene, Irgacure 784@BASF) dispersed PMMA (poly-[methyl methacrylate]) substrate system, with the aim of promoting the diffusion and polymerization processes in volume holographic storage. The two-wave interference system is adopted to record a permanent grating structure in our materials. The temporal diffraction variations of photopolymerization (during the interference exposure) and dark diffusion (after the interference exposure) processes have been investigated and analyzed. Aiming to analyze the influence of EY doping ratios on holographic performances, some key parameters were examined in the experiment. We first measured the temporal evolution of diffraction efficiency, then an exponential fitting was adopted to obtain the response time. Finally, the angular selectivity was evaluated by the Bragg condition after holographic recording. Also, the temporal evolution of each component is described by the nonlocal polymerization-driven diffusion model with a dual-photo-initiator composition, theoretically. Furthermore, we experimentally achieved the holographic grating enhancement in both the dark diffusion and photopolymerization processes by doping appropriate EY concentrations, respectively. This work provides a foundation for the acceptability of TI&EY/PMMA polymers in further holographic storage research.

## 1. Introduction

With the rapid development of the internet, the demand for timely information acquisition is gradually increasing [1]. Traditional magnetic and semiconductor storage technologies are limited by storage capacity and access rate [2,3]. In order to solve the technical obstacles in information storage, volume holographic storage technology emerged and has been continuously developed till now [4,5,6]. This technology is based on the interference and diffraction principles of electromagnetic waves to access information in the form of amplitude, phase, and polarization [7,8,9]. The information can be stored in phase gratings (refractive index modulated gratings) with a three-dimensional form, which can significantly increase the storage density. Meanwhile, multiple information pages can be accessed simultaneously by its unique optical-wave recording approach [10]. Therefore, the characteristics of volume holographic storage technology are high storage density, fast transmission rate, low manufacturing costs, and a simple preparation process, which can achieve mass output and meet the demands of current technological development. It is also considered the next-generation storage technology in the field of information access.

Holographic data storage reforms the way we write and retrieve data; this competitive technology is gradually entering the market with continuous research and practice [11]. The mainstream storage methods today are two-dimensional data storage, such as magnetic and semiconductor storage. Holographic storage can store information in volumetric form, significantly improving storage density [12]. Holographic storage has also been demonstrated to be a low-error storage method that guarantees the fidelity of data transmission [13]. Holographic storage can record information in the volume-phase grating, which is formed by the modulation of refractive index. Therefore, photorefractive materials are considered a very suitable recording medium. In recent years, photorefractive crystals [14] and photopolymer materials [15] have been widely studied. Compared with photorefractive crystals, photopolymers exhibit simpler preparation methods, lower preparation costs, and stable storage performance [15], which can be further researched and improved. Due to the unique advantages of holographic storage, it can be applied to the fields of three-dimensional display [16], holographic diagnosis [17], holographic sensors [18], etc.

In volume holographic storage, photo-initiators play a crucial role in the photopolymerization process, affecting the response time and grating intensity of the volume phase grating formation. In recent years, photopolymer materials have exhibited excellent information recording performance in volume holographic storage [19,20,21]. Materials adhered to PMMA and PVA substrates stand out among numerous photopolymer materials due to their unique advantages [22,23]. The PMMA-based materials exhibit excellent holographic storage density and information storage stability [24,25], while the PVA-based photopolymers display high storage efficiency and a fast response rate [26]. Different components in polymer materials contribute differently. As the substrate of the system, the monomer directly affects the physical properties of the material. At the same time, it can provide reactants in the process of photopolymerization. The photo-initiators mainly influence the response rate and grating strength during the photopolymerization process, which indicates they play a crucial role in the formation of volume-phase gratings. Many high-performance photo-initiated molecules have been discovered and examined, such as Phenanthrenequinone (PQ) molecules [27], Titanocene (Irgacure 784@BASF, TI) molecules [28], and Eosin Yellow (EY) molecules [29]. It is indicated that volume holographic performance can be effectively improved by adjusting the proportion and combination of photo-initiators.

In this work, we introduce additional photo-initiator EY molecules into the TI-dispersed PMMA substrate system. With this dual-photo-initiator doping approach, the material’s absorption efficiency for incident exposure can be improved during the holographic recording process. The nonlocal polymerization-driven diffusion (NPDD) model with a dual-photo-initiator composition is proposed to describe the consumption trend of TI and EY molecules during exposure. We investigated two main grating formation processes in holographic recording, i.e., the diffusion without additional exposure (dark diffusion) and the polymerization under continuous exposure (photopolymerization). In the experiment, we mainly studied the influence of EY doping ratios on holographic performances and kept the concentration of residual components unchanged. Some key holographic parameters, such as diffraction efficiency (DE), response time, and angular selectivity, have been measured to evaluate the holographic characteristics of TI&EY/PMMA polymers. Our research provides an experimental and theoretical basis for realizing holographic storage in a multi-photo-initiator dispersed PMMA-based photopolymer.

## 2. Materials and Methods

In this part, we introduce the main material preparation process and the optical measurement system in detail. The physical properties and the holographic parameters of our TI&EY/PMMA polymers would also be analyzed and defined here.

**Figure 1:**
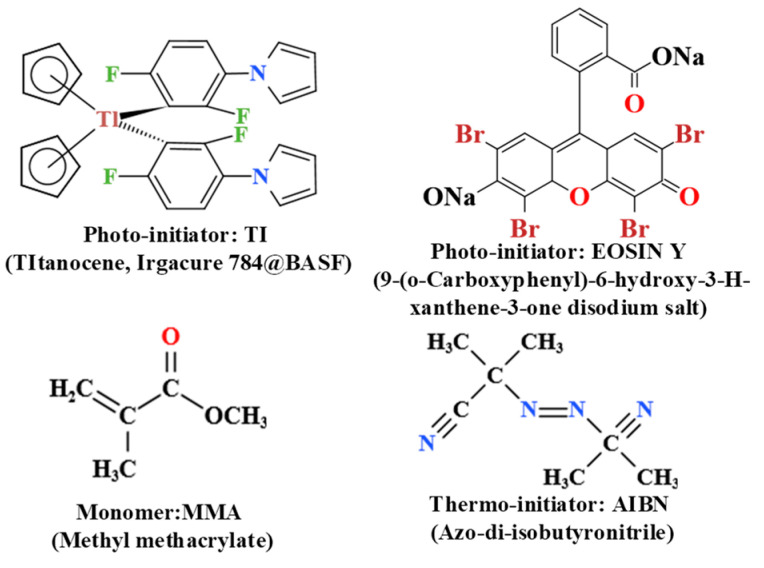
Chemical structures of the main ingredients in the TI&EY/PMMA material.

**Figure 2:**
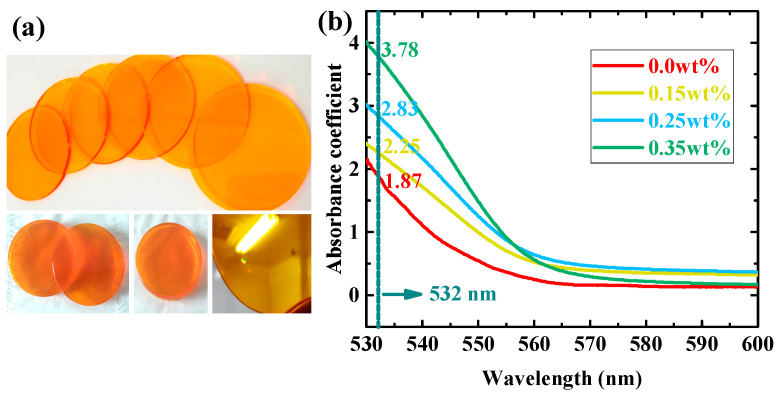
(**a**) TI&EY/PMMA samples prepared by our polymerization method; (**b**) the absorption spectrum of TI&EY/PMMA polymers in the range of 530–600 nm.

The TI&EY/PMMA polymers were prepared based on thermal polymerization methods [30]. The chemical structures adopted in the fabrication process are displayed in Figure 1 [31,32,33]. Traditional dye-dispersed PMMA photopolymer materials are prepared through thermal polymerization methods [30]. During this preparation process, the monomers MMA experience a thermal polymerization reaction under the catalysis of AIBN (azo-di-isobutyronitrile), resulting in the formation of long-chain PMMA photoproducts. This type of photoproduct exhibits excellent, stable physical properties and can be stored for a long time without the variation of internal structures. The incorporation of long-chain PMMA has been demonstrated in Ref [31], where the research offered several characterization methods to prove the existence of long-chain PMMA products in TI/PMMA polymers, such as the mass spectrum, the ^1^H-NMR spectrum, and the thermogravity analyses. By comparing the characterization results of materials before and after exposure, the corresponding molecular weight of PMMA can be observed. Also, another study characterized the FTIR spectrum and SEM image before and after exposure to PMMA-based photopolymers and confirmed that the long-chain PMMA polymers would be produced by photopolymerization [4]. It is worth noting that dye molecules do not participate in the reaction during this process; they only exist as an introduced component. Dye molecules are also known as photo-initiators due to their photosensitive properties. Therefore, unreacted photo-initiators can play a dominant role in the holographic recording process. For PMMA polymer systems, the role of AIBN is to promote thermal polymerization reactions of monomers under heating, thereby regulating the thickness and size of the material. The role of the photo-initiator is to promote the photopolymerization reaction of monomers under exposure, thereby regulating the refractive index and forming holographic gratings. As for our TI&EY/PMMA polymers, the detailed fabrication processes are as follows: The TI and AIBN (azo-di-isobutyronitrile) molecules with optimized doping ratios (TI molecules: 4.0 wt%; AIBN molecules: 2.0 wt%) were dissolved into the substrate (MMA monomers) by magnetic stirring. The mixture could be completely homogenous after 12 h of stirring at 40 °C. Then, another photo-initiator, EY molecules, were mixed into the viscous liquid for an additional 12 h of stirring in the same experimental surroundings. The reason for using magnetic stirring was that it could evenly dissolve various components in the solvent. After the two photo-initiators were uniformly distributed on the substrate, we began the curing process. The mixture would be transferred to an incubator for curing, which could be divided into two steps, i.e., rapid polymerization and low-temperature polymerization. In the first stage, the temperature was set to 72 °C for 15 min, while the second was 45 °C for 48 h. The purpose of using two-stage heating was to sufficiently excite AIBN molecules while slowing down the thermal polymerization duration of monomers. We obtained solidified polymer materials with good transparency after the curing process, which could be used for holographic storage measurements, as shown in Figure 2a. The size of our sample is six centimeters in diameter and 2 mm in thickness.

In the fabrication process, we adopted four doping ratios of EY molecules, i.e., 0.0 wt%, 0.15 wt%, 0.25 wt%, and 0.35 wt%. Firstly, we evaluated the photosensitive properties of our material by examining the absorption spectrum in the range of 530–600 nm, as depicted in Figure 2b. By doping EY molecules, the absorption coefficient at 532 nm could be increased by more than 2-fold, from 1.87 to 3.78, which indicated the absorption efficiency of the TI/PMMA polymer system would be effectively developed in the visible light band by introducing the EY molecules. This material has a peak absorption coefficient in the ultraviolet band, but an excessive absorption coefficient can also lead to a strong holographic scattering phenomenon. Furthermore, it is difficult to establish the holographic optical platform by using ultraviolet light as the exposure source. Therefore, a 532 nm light source was adopted in the holographic recording and reconstructing process, which not only meets the material’s absorption efficiency towards exposure but also avoids the influence of excessive holographic scattering on the grating formation process.

**Figure 3:**
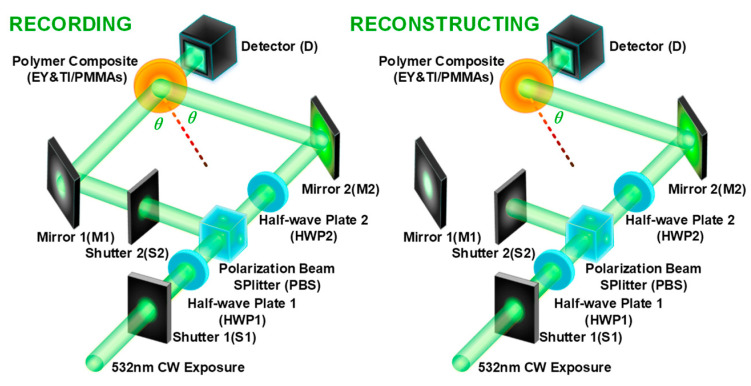
Recording and reconstructing system for volume holographic storage.

Volume-holographic storage technology utilizes the principles of optical interference and diffraction. The premise for interference between two exposure beams is that they possess the same exposure energy, a constant phase difference, and the same polarization direction. When the two exposure beams encounter, interference fringes of bright and dark intersections will be generated. If the holographic recording medium is sensitive to exposure intensity, the interference fringes induced by optical interference will be recorded inside the medium as variations in refractive index, which will finally form the holographic interference gratings. In the process of information storage, the required data is loaded onto the wavefront of the incident beams, and it can be recorded in the form of amplitude, polarization, and phase into the volume holographic gratings by optical interference principles. In our experiment, we also assembled the holographic recording system based on the principle of two-wave coupling interference. The recording and reconstructing processes of holographic gratings were separated, as shown in Figure 3. Firstly, shutter 1 (S1) and shutter 2 (S2) were opened to make an intersection inside the recording medium of two exposure beams, which were split by the polarization beam splitter (PBS). The half-wave plate 1 (HWP1) could adjust the exposure intensity of two separate beams, while the half-wave plate 2 (HWP2) could regulate the polarization state of one exposure beam. It is possible to separate the polarization and amplitude regulation of incident beams by introducing the PBS, which makes the entire recording system more flexible. Two separated beams regulated by HWP1 and HWP2 could generate interference fringes inside the material, eventually forming the volume holographic grating. Secondly, the DE of holographic gratings could be readout by the reconstructing apparatus, as shown in Figure 3. In this step, the S2 was closed to prevent further enhancement of grating strength. We illuminated the holographic grating with a beam along the same optical path as the recording process to obtain its diffraction intensity. In the experiment, two main processes, the dark diffusion enhancement process (DDEP) and the continuous exposure enhancement process (CEEP), were detected by this approach. The dark diffusion process refers to a continuous refractive index variation in the exposure area of the recording medium without additional holographic interference exposure, thereby affecting the strength of the holographic grating. The main reason for this process is that the exposure area is divided into two parts: the bright and the dark regions. The photo-initiators in the bright region can absorb photons to polymerize with the surrounding monomers, while the photo-initiators in the dark region cannot be consumed due to the lack of exposure. This leads to a concentration difference of photo-initiator molecules between the bright and dark regions after holographic interference exposure, which in turn causes a diffusion process along the concentration gradient of the photo-initiator molecules. It indicates that the holographic grating strength under dark diffusion is modulated by the gradient diffusion of photo-initiators. The continuous exposure process refers to the variation trend of holographic grating strength with the accumulation of exposure energy. During this process, the photo-initiator molecules in the bright region are continuously consumed, leading to a gradual increment in the concentration difference of photo-initiator molecules between the bright and dark regions, which will also cause a diffusion process along the concentration gradient. It implies that the holographic grating strength is influenced by the polymerization and diffusion processes of photo-initiator molecules during continuous exposure. These two processes (DDEP and CEEP) can judge the holographic performances of our photopolymer samples, which will be manipulated in our experiment.

The scheme of the first process was to examine the temporal growth of holographic diffractions in dark conditions after 30 s of illumination. The second aimed at investigating the real-time growth trend of holographic diffraction under continuous exposure lasting for 100 s. A 532 nm green beam solid-state laser was chosen to provide sufficient illumination energy without excessive holographic scattering. The exposure intensity during the holographic recording was set to 115 mW/cm^2^. The DE and the response time were two main indexes used to evaluate the holographic performances. The definition equation of temporal diffraction efficiency (DE(t)) in the experiment is depicted as follows:

(1) $$\mathrm{DE}\left(t\right)=I_{d}(t)/I_{i}(t)$$

where $I_{d}(t)$ and $I_{i}(t)$ describe the temporal exposure intensity of diffracted and incident beams. The diffraction efficiency directly reflects the strength of the holographic gratings in the experiment. Hence, the grating strength (GS(t)) can be defined as the square root of diffraction efficiency (DE(t)),

(2) $$\mathrm{GS}\left(t\right)=\sqrt{\mathrm{DE}\left(t\right)}$$

As we mentioned above, the main reason for the formation of holographic gratings is due to the refractive index modulation induced by the photopolymerization and diffusion processes. Therefore, the diffraction efficiency is also related to the refractive index modulation in theory. According to Kogelnik’s theory [34], we can get the relation equation between diffraction efficiency (DE(t)) and refractive index modulation (Δn(t)),

(3) $$\mathrm{DE}(t)=Τ\sin^{2}\left(\frac{\Delta n(t)\pi d}{\lambda \cos\theta }\right)$$

where Τ stands for the absorption, scattering, and reflection losses factor; d depicts the material thickness; and λ and θ describe the wavelength and intersection angle of incident beams, respectively. In the Kogelnik theory, the absorption grating model is separated from the dielectric grating model. In the absorption-grating model, the refractive index modulation of the material is zero. Though the material has an absorption efficiency on exposure, the main reason for the holographic grating formation is the variation of refractive index. Hence, the results of the dielectric grating model are adopted to describe the relation between diffraction efficiency and refractive modulation index. At this point, we have connected the experiment and theory using the parameter of diffraction efficiency (DE(t)). Another key parameter, the response time τ, was defined as the exponential fitting coefficient of temporal diffraction efficiency evolution curves, as shown in Equation (4). DE(t) and ${\mathrm{DE}}_{\max}$ depicted the temporal evolution and maximum value of volume holographic diffraction efficiency while t described the exposure durations. Due to the response time τ is obtained by fitting the growth trend of diffraction efficiency, it can reflect the formation speed of holographic gratings in our photopolymer materials during the continuous exposure process. In the experiment, the diffraction efficiency and the response time mainly evaluated the strength and storage rate of holographic gratings.

(4) DE(t)=DEmax[1−exp(−tτ)]

## 3. Results and Discussion

### 3.1. Dual-Photo-Initiator Doping NPDD Model

A NPDD model-based dual-photo-initiator doping process is proposed, aiming at describing the photophysical chemistry progress internal to the TI&EY/PMMA polymers [35,36]. The main reaction mechanism of photopolymerization is the initiation and polymerization of internal photo-initiators, as well as the promotion of crosslinking between monomers. Based on the TI-molecule doping system, the introduction of EY molecules can provide additional absorption of exposure energy, thereby providing more free radicals to generate chain polymerization reactions and eventually increasing the stability of grating strength. The specific photochemical reactions during the exposure process are as follows:

(5) $$\mathrm{TI}+h\nu \overset{k_{TI}}{⇄}\left\{ \begin{array}{l} {\left[TI\right]}^{*}+\mathrm{PMMA}\Rightarrow \mathrm{TI}\text{-}n\mathrm{MMA}\\ {\left[TI\right]}^{*}+h\nu \Rightarrow {\mathrm{TI}}^{˙˙}+2\mathrm{PMMA}\Rightarrow n\mathrm{MMA}\text{-}\mathrm{TI}\text{-}n\mathrm{MMA} \end{array}\right.$$

**Figure 4:**
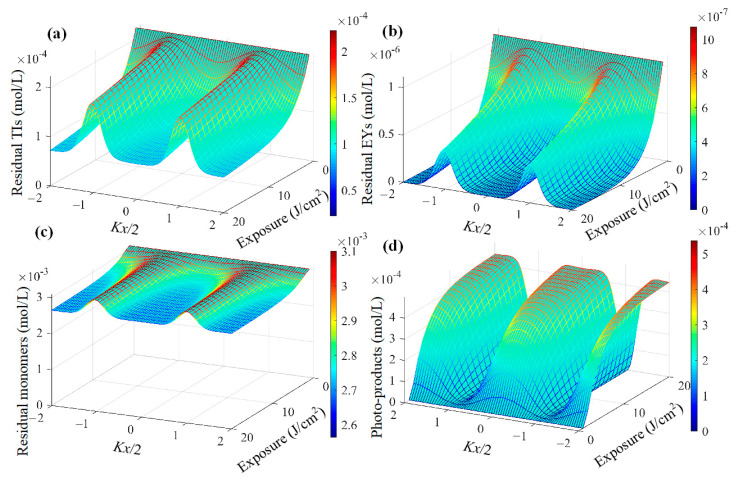
Theoretical evolutions of each component concentration during exposure: (**a**) TI molecules, (**b**) EY molecules, (**c**) monomers, and (**d**) photo-products.

(6) EY+hν→kEY[EY]3+PMMA⇒[EY]*+[PMMA]*⇒EY-PMMA

where TI and EY represent the initial concentration of TI and EY molecules, PMMA and ${\left[\mathrm{PMMA}\right]}^{*}$ stand for the concentration of monomers and production of free radicals. ${\left[TI\right]}^{*}$ and ${\mathrm{TI}}^{˙˙}$ describes the excitons and cations of TI, while [EY]3 and ${\left[\mathrm{EY}\right]}^{*}$ depicts the triplet and singlet excitons of EY, respectively. Two photo-initiators exhibit different polymerizations during exposure. The photo-cleavage of TI molecules can be initiated by exposure, which can generate TI cations to polymerize with monomers. EY molecules can be transitioned to an excited state after absorbing photons and directly polymerize with surrounding monomers to form the photo-product. By introducing the non-local response function: $R(x,x’)=\frac{1}{\sqrt{2\pi \sigma }}\exp\left[\frac{-{\left(x-x’\right)}^{2}}{2\sigma }\right]$ [36], the diffusion process of the photo-initiator during exposure can also be described. The parameter σ represents the length of non-localized polymer chains, which can reflect the multi-level reactions of monomer chain polymerization. According to the NPDD model, we can obtain the evolution of component concentrations with the increment of exposure durations, as shown below:

(7) $$\frac{\partial [TI](x,t)}{\partial t}=\frac{\partial }{\partial x}D_{\mathrm{TI}}\frac{\partial [TI](x,t)}{\partial x}-\int_{-\infty }^{+\infty }R(x,x’)P_{TI}(x,x’)dx’$$

(8) $$\frac{\partial [\mathrm{EY}](x,t)}{\partial t}=\frac{\partial }{\partial x}D_{\mathrm{EY}}\frac{\partial [\mathrm{EY}](x,t)}{\partial x}-\int_{-\infty }^{+\infty }R(x,x’)P_{\mathrm{EY}}(x,x’)dx’$$

(9) $$\frac{\partial [\mathrm{MMA}](x,t)}{\partial t}=\frac{\partial }{\partial x}D_{\mathrm{MMA}}\frac{\partial [\mathrm{MMA}](x,t)}{\partial x}-\int_{-\infty }^{+\infty }R(x,x’)\left[P_{\mathrm{TI}}(x,x’)+P_{\mathrm{EY}}(x,x’)\right]dx’$$

(10) $$\frac{\partial [\mathrm{Product}](x,t)}{\partial t}=\int_{-\infty }^{+\infty }R(x,x’)\left[k_{\mathrm{TI}}[TI](t)+k_{\mathrm{EY}}[EY](t)\right]dx’$$

where *D* and *P* represent the diffusion and polymerization rates of the corresponding component; $k_{\mathrm{TI}}$ and $k_{\mathrm{EY}}$ stand for the initiation rates of TI and EY molecules, respectively. Specific parameters can be referred to in refs [26,35,36]. The theoretical diffused-polymerization process of TI&EY/PMMA polymers during exposure is displayed in Figure 4. The initial concentrations of TI, EY, and MMA molecules are 2.234 × 10^−4^, 0.1 × 10^−4^, and 3.1 × 10^−3^ mol/m^3^, respectively, which are determined based on the experimental doping content. From the simulations we can see, the slight doping amount of EY molecules is completely reacted with during the exposure process, which provides additional excitons for polymerization reactions. This process ultimately promotes grating formation and reinforces grating stability. We experimentally investigated and verified whether the dual-photo-initiator system can enhance the holographic grating formation in dark diffusion and photopolymerization processes.

### 3.2. Experimental Investigations of Holographic Performances in TI&EY/PMMA Polymers

In the experiment, we mainly investigate the influence of EY doping ratios on holographic performances in TI&EY/PMMA polymers. Hence, the concentration of another photo-initiator, TI molecules, has been determined (TI doping ratio: 4.0 wt%). Four doping ratios of EY molecules have been adopted, i.e., 0.0 wt%, 0.15 wt%, 0.25 wt%, and 0.35 wt%, respectively. A two-stage experimental measurement process has been executed to evaluate the holographic performances, namely separated recording and reconstructing steps, as shown in Figure 3. We record holographic gratings generated by the interference of two incident beams (objects and reference waves) to form alternating bright and dark stripes. We used another reconstructing beam with the same optical path as the reference wave to read out the holographic grating strength based on the diffraction principle. Two key processes in volume holographic storage, dark diffusion and photopolymerization, are measured and analyzed in detail.

**Figure 5:**
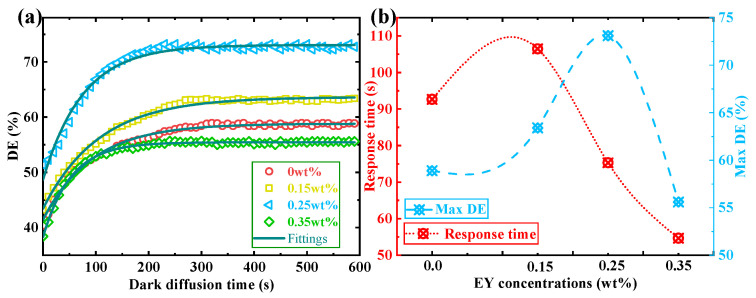
(**a**) Temporal evolution of DE in the DDEP with different EY doping ratios; (**b**) the response time and max DE in the DDEP with different EY doping ratios.

During the dark diffusion process, we pre-expose our TI&EY/PMMA polymers for 30 s in order to generate a holographic grating inside the sample. Then, we keep the material in the darkness without additional interference. The DE of the holographic grating is readout every 10 s by our reconstructing assembly. It is notable that the exposure energy during the reconstructing process is weak enough so that it will not cause excessive attenuation of the holographic grating strength. The dark diffusion evolution of DE has been measured by this procedure, as shown in Figure 5a. The essence of dark diffusion is the forward gradient diffusion process of photo-initiator molecules after interference exposure. During the exposure, the interference occurs within the material due to the coupling of incident beams, resulting in alternating bright and dark regions in the exposure area. In the bright region, photo-initiator molecules are sufficiently consumed, while in the dark, photo-initiators are barely consumed. From an optical perspective, the dark diffusion is caused by the concentration difference of photo-initiators during the interference process. After suspending the exposure, the concentration discrepancy of photo-initiators is no longer enlarged. Photo-initiator molecules gradually diffuse from the dark to the bright region due to this concentration difference, further modulating the refractive index of the bright and dark regions. Hence, the holographic grating strength can be enhanced by increasing the modulation depth of the refractive index. The dark diffusion process can provide an effective and automatic approach to enhancing the grating strength in holographic storage. The introduction of this process can reduce the duration of interference exposure, which can reduce the holographic scattering caused by long-term exposure and improve the quality of recorded information. In addition, it conserves more energy and improves the efficiency of exposure utilization, which benefits the long-term, stable storage of information. These are all potential application directions of the dark diffusion in volume holographic storage, which are mostly implemented to optimize the storage performance.

The experiment results indicate that the introduction of EY molecules promotes the dark diffusion effect. The DE increment and the response time of dark diffusion will be improved, as shown in Figure 5b. After doping the EY molecules, the maximum optimization of DE and response time in the DDEP are 24.1% (EY doping ratio: 0.25 wt%) and 41.0% (EY doping ratio: 0.35 wt%), respectively. The main reason for this result is that the absorption efficiency of exposure energy can be enhanced by adding an additional photo-initiator, thereby improving the progress of diffusion and polymerization.

**Figure 6:**
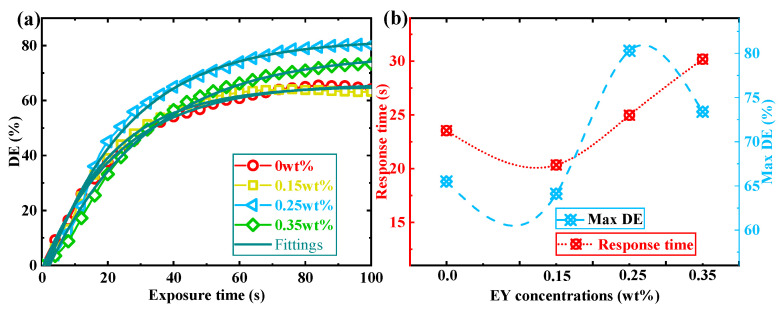
(**a**) Temporal evolution of DE in the CEEP with different EY doping ratios; (**b**) the response time and max DE in the CEEP with different EY doping ratios.

Furthermore, we also investigate the influence of EY doping ratios on the real-time grating enhancement process under continuous exposure. In this stage, we maintain a periodic operation of S2. During each circle, the shutter is opened for 4 s to generate interference fringes and closed for 0.5 s to readout the grating strength. The temporal evolution of DE in TI&EY/PMMA polymers is measured with 100 s of continuous exposure to different EY doping ratios, as shown in Figure 6a. To make a direct comparison, the response time and max DE with different EY doping ratios are depicted in Figure 6b, where the response time can be optimized by 13.6% (EY doping ratio: 0.15 wt%) and max DE can be enhanced by 22.3% (EY doping ratio: 0.25 wt%). After a comprehensive evaluation of the above two processes, the holographic performances can be improved by doping EY molecules into the TI/PMMA polymer system. The max DE under the DDEP and CEEP are both significantly enhanced, with a slight difference in response time. A more suitable EY doping ratio is determined by 0.25 wt%, where the DE in the DDEP is improved from 58.9% to 73.1% and the DE in the CEEP is enhanced from 65.5% to 80.3%.

**Figure 7:**
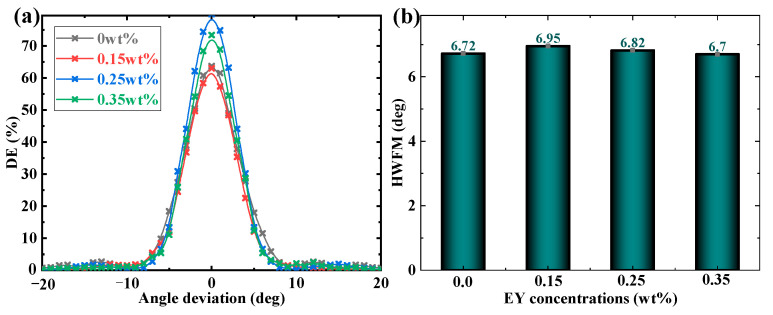
(**a**) The evolution of angular selectivity in TI&EY/PMMA polymers with different EY doping ratios; (**b**) the FWHM in TI&EY/PMMA polymers with different EY doping ratios.

The capacity of angular-multiplexing is a key element in evaluating the storage density of our recording medium. The multiplexing ability can be enhanced by shortening the Bragg angle. Hence, we examined the angular selectivity of TI&EY/PMMA polymers with different EY doping ratios, as shown in Figure 7. The full width at half-maximum (FWHM) is adopted to estimate the angular-multiplexing capacity of recording materials [37]. A smaller value of FWHM can contribute to a higher storage capacity by using angular-multiplexing technology. The experiment results indicate that the variation of EY doping ratios has a neglectable influence on the HWFM, as shown in Figure 7b. Therefore, on the premise of improving the holographic performance, doping EY molecules will not influence the multiplexing storage capacity of the TI/PMMA polymer system. It implies that the TI&EY/PMMA polymers can be adopted as a potential photopolymer material to proceed with further holographic investigations and analyses.

To analyze the improvement of dispersing EY molecules into TI/PMMA polymers, some relevant works in the field of holographic storage have been collected and compared, as shown in Table 1. We mainly focus on the parameters of diffraction efficiency (DE) and response time (τ). It is indicated that the holographic performance can be significantly improved in TI/PMMA polymers by doping EY molecules. We achieved over 80% diffraction efficiency and maintained a response time of over 20 s. This type of material, with its fast photosensitivity and high grating strength, exhibits strong competitiveness in the field of data storage.

Holographic storage is a data access approach with three-dimensional form internal to the recording medium by laser exposure. Compared to traditional storage methods, the advantage of holographic storage is that it can store massive amounts of data in units of volume and achieve permanent preservation of information. Also, it exhibits fast read and write rates, which significantly improves storage efficiency. Additionally, the accessing process is not affected by environmental factors such as humidity and temperature, resulting in a higher fidelity and signal-to-noise ratio. These characteristics make it widely applicable in various fields, such as holographic elements [42], lasers [43], and holographic sensors [44]. At the same time, in the field of cloud computing [45], the introduction of holographic storage can significantly improve the cloud space and make the operation of data more flexible. In terms of artificial intelligence, holographic storage can also provide a more convenient storage method for deep learning databases [46]. In summary, the application prospects of holographic storage are very broad, and they will lead us into a more efficient, fast, and secure information age.

## 4. Conclusions

With the rapid development of digital time, the demand for large-capacity, fast, and convenient storage technology is growing. Volume-holographic storage can be regarded as the next-generation storage technology for applications due to its unique characteristics. In this paper, we introduce an additional photo-initiator, EY molecules, into the TI/PMMA polymer system and prepare a dual-photo-initiator photopolymer material, namely TI&EY/PMMA polymer. Two main processes of volume holographic storage, dark diffusion and photopolymerization, have been examined by two-wave coupling interference equipment. The experimental results indicate that the holographic performances of TI/PMMA polymers can be effectively improved by doping EY molecules. An optimized EY doping ratio of 0.25 wt% is obtained. The diffraction efficiency is improved from 58.9% to 73.1% in the dark diffusion process, while it is enhanced from 65.5% to 80.3% in the photopolymerization process with continuous exposure. Also, the nonlocal polymerization-driven diffusion model with a dual-photo-initiator composition is proposed to describe the internal component variations of TI&EY/PMMA polymers, which implies that doping EY molecules can effectively enhance the absorption efficiency of materials with consecutive exposure, thereby promoting the diffusion and polymerization processes. Our research provides a necessary experimental and theoretical basis for the further application of TI&EY/PMMA polymers.

## Figures and Tables

**Table 1:** Comparisons of holographic parameters on PMMA-based photopolymers in relevant research fields.

Photopolymer	Thickness	DE	τ
PQ/PMMA [38]	1 mm	2%	around 150 s
PQ/PMMA [39]	1.5 mm	62.6%	41.8 s
PQ/PMMA [40]	3 mm	48%	31 s
TI/PMMA [41]	1 mm	52%	30 s
TI/PMMA [5]	2 mm	65.5%	23.5 s
EY&TI/PMMA	2 mm	80.3%	24.9 s

## Data Availability

The data presented in this study are available on request from the corresponding authors.
